# The changing shape of the ISCEV standard pattern onset VEP

**DOI:** 10.1007/s10633-017-9596-8

**Published:** 2017-06-13

**Authors:** Dorothy A. Thompson, Dennis M. Fritsch, Sharon E. Hardy, The POW Study Group

**Affiliations:** 1 The Tony Kriss Visual Electrophysiology Unit, Clinical and Academic Department of Ophthalmology, Great Ormond Street Hospital for Children, Great Ormond Street, London, WC1N 3JH UK; 2 UCL Great Ormond Street Institute of Child Health , 30 Guilford Street, London, WC1N 1EH UK

**Keywords:** Pattern onset VEP, ISCEV standard VEP waveform, Waveform maturation, Check size, Age

## Abstract

**Purpose:**

Pattern onset VEPs do not always show distinct C1–C2–C3 peaks and troughs. Our purpose was to study changes in pattern onset VEP with age to determine when the illustrated ISCEV standard onset VEP waveform can be reliably recorded.

**Methods:**

We recorded pattern onset VEPs from an Oz electrode referred to mid-frontal electrode according to ISCEV standards by presenting checks of 60′ and 15′ side length in a 15° field. Twenty-four adults aged 20–63 years participated. Amplitudes and latencies were collated. Pattern onset adult VEP shapes were compared to the waveform published in the ISCEV VEP standard and to paediatric pattern onset VEP waveforms recorded from 16 infants aged 7 months.

**Results:**

The shape of the pattern onset VEP changed gradually with age. The C1–C2–C3 morphology of the ISCEV standard pattern onset VEP becomes apparent consistently after 40 years to 60′ check stimulation. As age increases a negative trough, C2 is more frequently seen; however, the broad positive peak which characterises infant onset VEPs may still be recorded at 20 years. The group median measurements of onset VEPs to 60′ were C1 7 µV@ 88 ms (range 67–110 ms), C2 9 µV@109 ms (range 89–158 ms) and C3 13 µV@121–246 ms. To smaller 15′ checks, peak latencies were earlier and C2 became more obvious. The group median measures of onset VEPs to 15′ were C1 2 µV@69 ms (55–108 ms), C2 10 µV@90 ms (77–145 ms) and C3 14 µV@122 ms (99–200 ms).

**Conclusion:**

The ISCEV standard onset VEP best describes the waveform configuration and latency of the onset VEP produced by 60′ checks in adults of more than 40 years of age. The onset VEP waveform produced by 15′ checks is distinguished by more prominent negative C2 and earlier C1 and C2 latencies.

## Introduction

Three visual stimuli are described in the 2016 ISCEV VEP standard; pattern reversal, pattern onset and flash [1]. Pattern reversal stimulation is the gold standard. A phase-reversing draughtboard produces a pattern reversal VEP which is characterised by a positive peak at a latency of 100 ms. This is established by 7 months of age and is highly reproducible across individuals [2, 3]. Pattern onset and flash stimulation are recommended for patients with active defocus or nystagmus, or to identify chiasmal misrouting in albinism. VEPs produced by pattern onset and flash stimulation have complex polyphasic waveforms and show considerable inter-individual variation [1].

The pattern onset VEP waveform shown in the ISCEV VEP standards has well-described C1-positive–C2-negative–C3-positive peaks [1], but in practice these individual peaks are not always identifiable. Infants, for example, tend to show a single, simplified broad positive peak, which becomes more complex with maturation [4]. There are few published examples of pattern onset VEP waveforms. We sought to better describe and understand the waveform changes that may be expected in pattern onset VEPs produced by different check sizes at different ages when the ISCEV standard protocol is used.

## Methods and subjects

A cross-sectional observational study was carried out. Pattern onset VEPs were elicited from 24 adult subjects aged 20–63 years to the ISCEV standard VEP protocol which stipulates check side lengths 60′ and 15′ presented in a minimum 15° field recorded from Oz referred to Fz. The stimuli were presented for 200 ms onset/followed by 400 ms offset of mean luminance 82 cd/m^2^ on a plasma display panel Michelson contrast 96% (max 170 cd/m^2^/min 6 cd/m^2^) viewed at 1 m. Pattern onset VEPs were additionally recorded from 5 adults who viewed the same stimuli in a larger 30° field and with a shorter onset period of 200 ms and from 10 teenagers to 60′ checks presented with an additional, longer inter-stimulus interval/offset interval of 1000 ms

Pattern onset VEPs recorded from Oz-mf 16 infants aged 7 months were retrospectively reviewed from a sample of more than 200 infants who were tested within the first year of life when laboratory reference data were compiled. The age 7 months was selected because it is the age at which pattern reversal p100 latencies fall within 10% of adult values. Onset VEPs in this infant reference study typically were recorded using a wider range of check sizes, 400′, 200′, 100′, 50′, 25′ and 12.5′ presented for 230 ms in a 30° field followed by a field of mean luminance for 330 ms. For this study, the stimuli had been displayed on a 74-cm NEC multi-synchronisation monitor (contrast 80% and luminance 50 cd/m^2^).

 The acquisition trigger timing, field size and check sizes presented on the plasma display panel were adjusted to match the NEC monitor. All data were recorded using the Espion system. The EEG was digitised using a sampling rate of 1 kHz and a band-pass filter of 0.312–100 Hz. The amplifiers had a fixed gain with an input range of ±0.5 V (Espion by Diagnosys, Cambridge, UK). The impedances of all electrodes were balanced and maintained below 5 kΩ throughout the recordings. In all cases, central fixation was monitored by CCTV.

The onset VEP waveforms were evaluated and the amplitude and latency of the main peaks and troughs measured. In cases where C1–C2–C3 morphology was not defined, the first main positive peak was taken as C1. In cases where C1 was not identified, but a negative C2 was prominent, the time point at the beginning of the C2 descent from baseline was taken as a C1 latency.

## Results

**Fig. 1:**
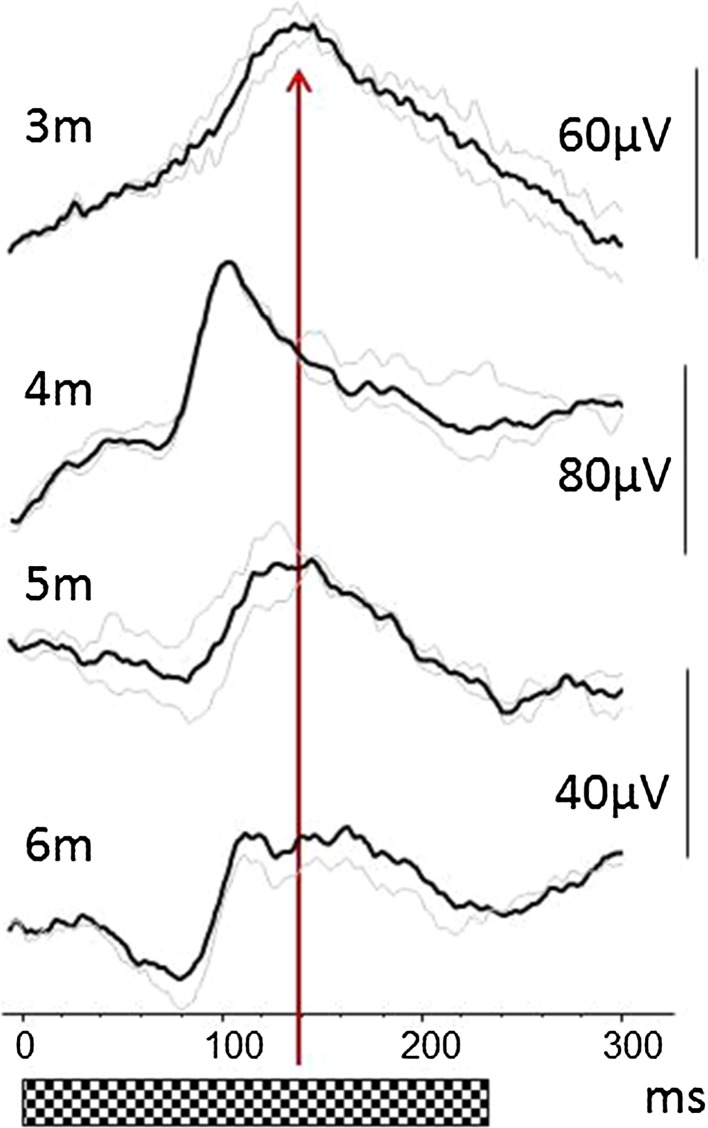
Example pattern onset VEPs waveforms from infants aged 3, 4, 5 and 6 months show a trend for the positive peak latency to reduce from 140 to 100 ms. The *solid line* is the average, repeated trials are shown in *grey*

**Fig. 2:**
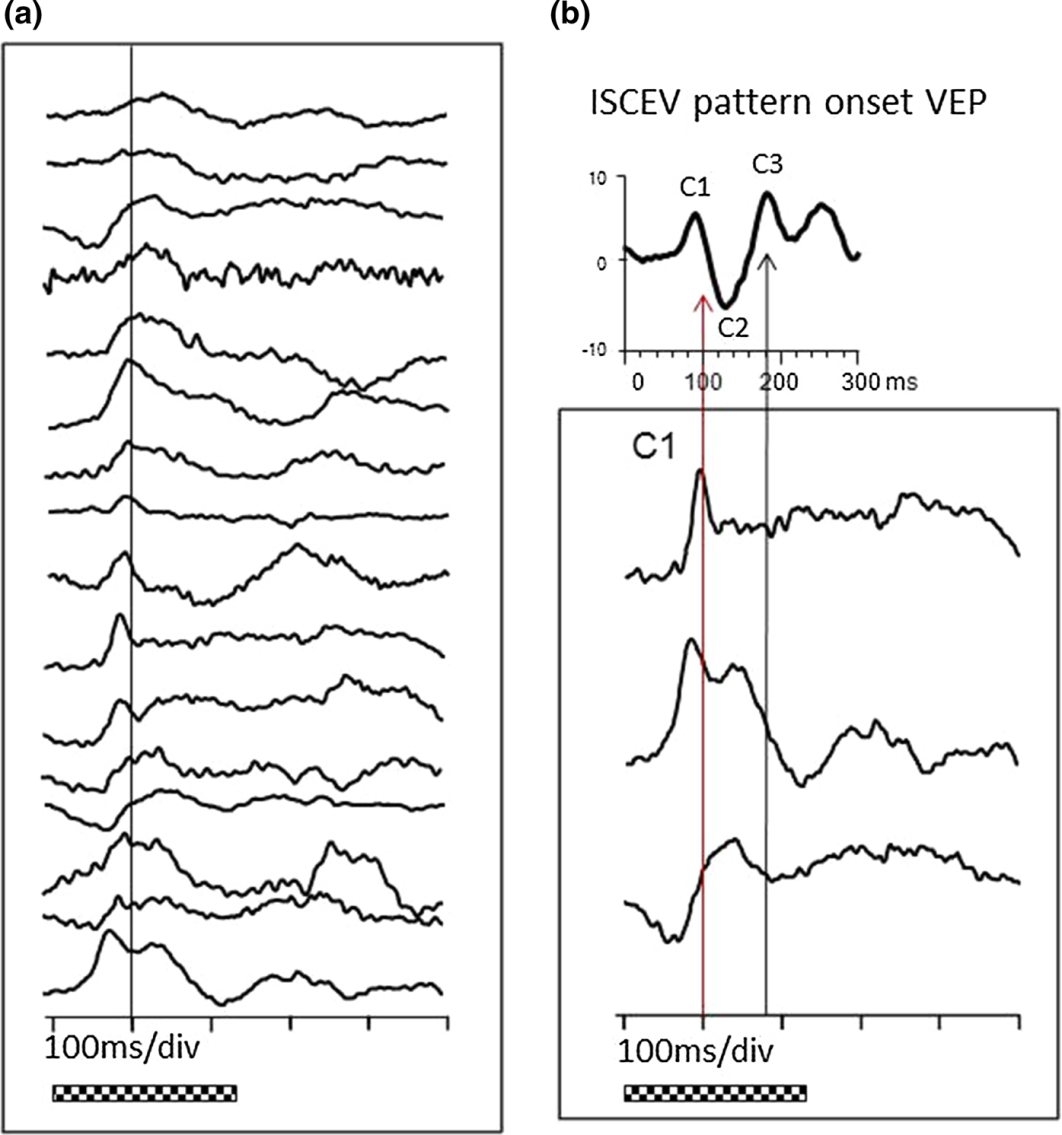
**a** Average onset waveforms from 16 individual infants all aged 7 months are arrayed. **b** Waveforms from 3 infants that exemplify the maximal waveform variation at 7 months are highlighted. The ISCEV standard waveform is shown on the same timescale in *black* for ease of comparison above

Pattern onset waveforms examples from healthy infants are shown in Fig. 1. Data from 16 infants aged 7 months, the age at which reversal VEPs are within 10% of adult latency [3], are shown in Fig. 2a, b.

At 7 months 13/16, infants showed an early positive peak mean C1 13 µV@94 ms (range 76–112 ms), seen in the top Fig. 2b. Of 13, 5 showed only the early positive peak, but 8/13 showed a positive peak at 138 ms as well (middle trace Fig. 2b). Of 16, 3 infants showed only a later positive peak (possibly C3), mean 135 ms (range 130–138 ms) bottom trace Fig. 2b.

**Fig. 3:**
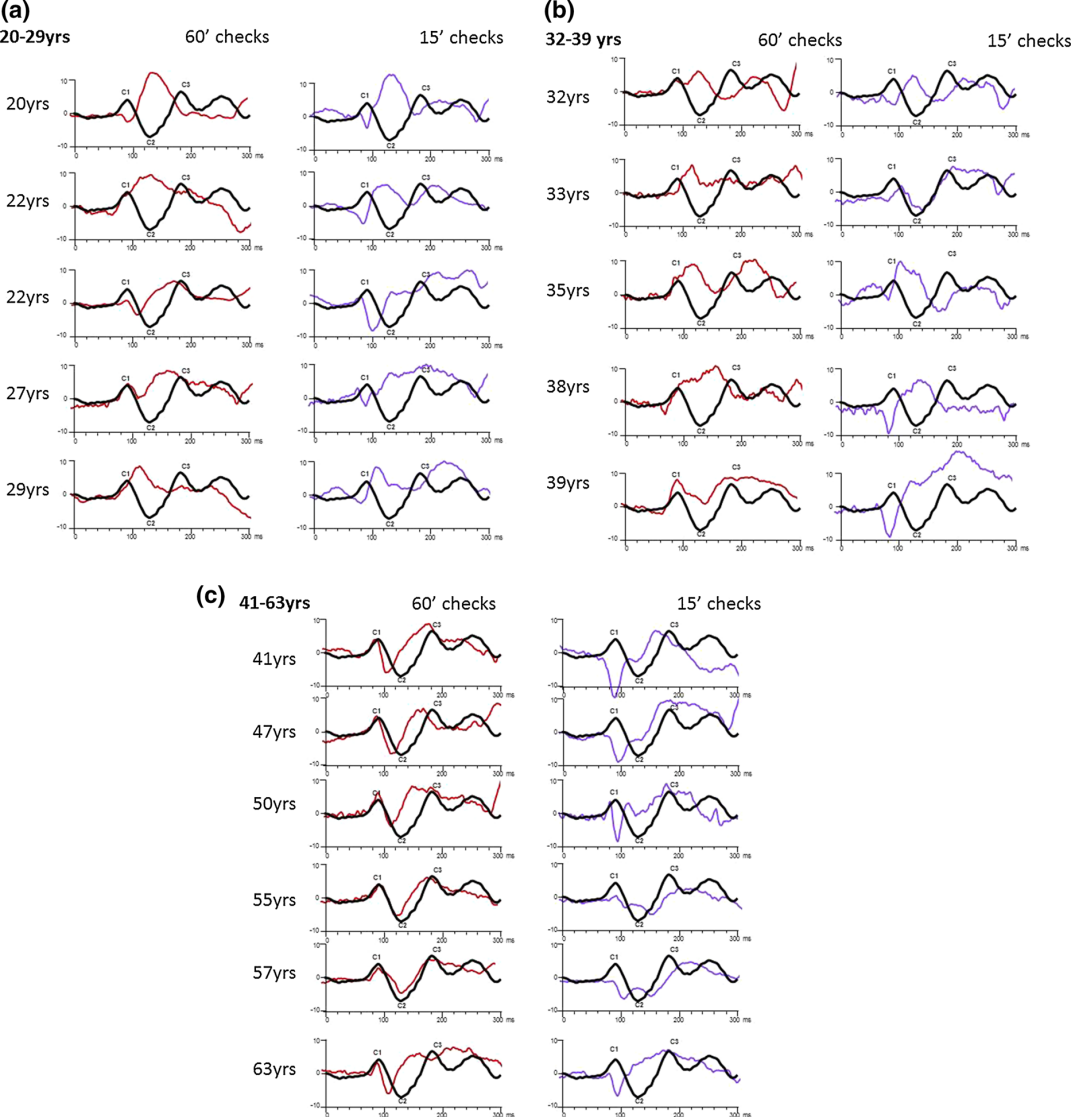
Three panels of adult pattern onset trace arrays are arranged in decades (**a**–**c**). Onset VEP waveforms produced by 60′ and 15′ checks in the same individual are displayed alongside each other, and each one is superimposed on the ISCEV template waveform in *black* for comparison. **a** Adult (aged 20–29 yrs), pattern onset waveforms overlaid on ISCEV standard waveform. **b** Adult (aged 32–39 yrs), pattern onset waveforms overlaid with ISCEV standard. **c** Adult (aged 41–63 yrs), pattern onset waveforms overlaid with ISCEV standard

**Fig. 4:**
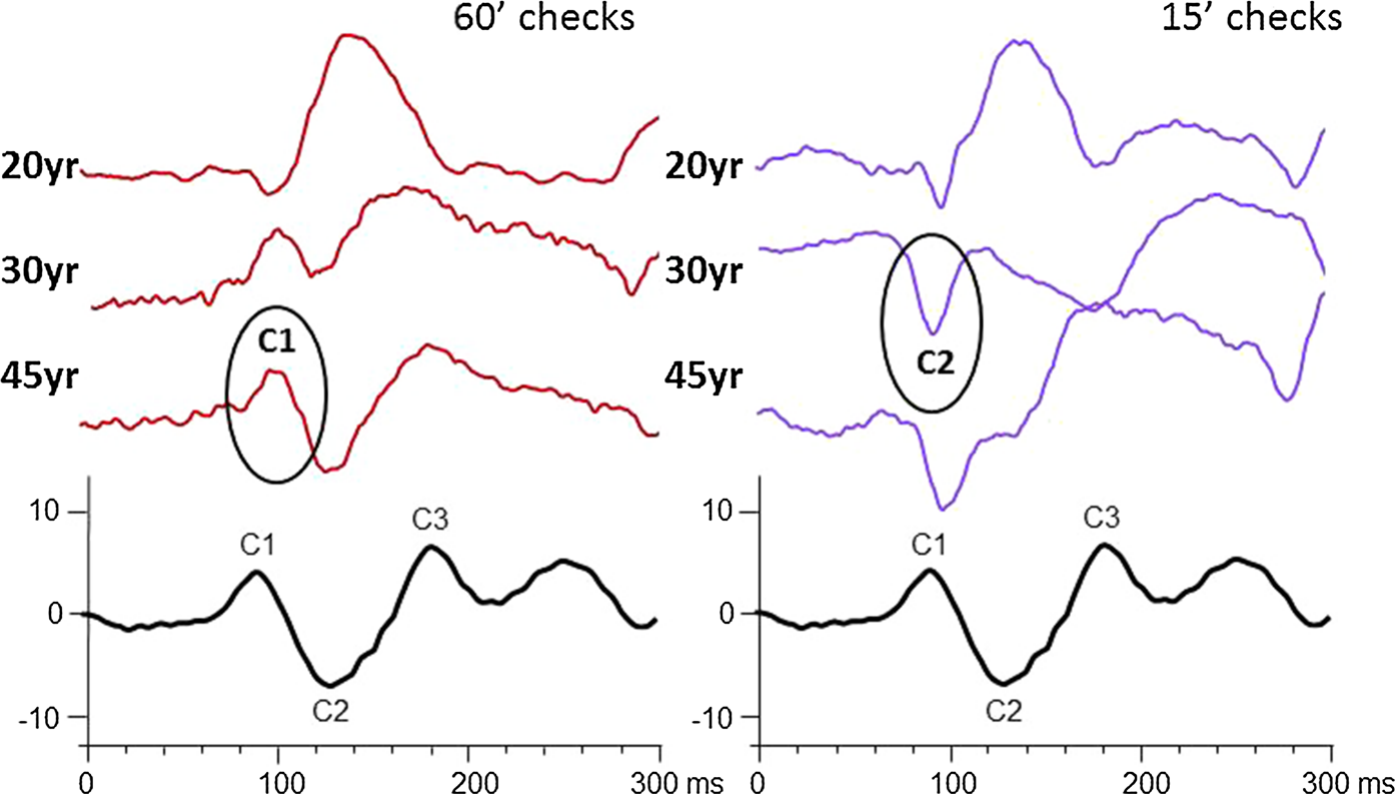
Example waveforms from 3 individuals are shown that summarise the main change in waveform features with check size and age during adulthood

Adult pattern onset VEP waveforms are arrayed in broad decade panels in Fig. 3. The ISCEV standard pattern onset VEP C1–C2–C3 configuration is seen more consistently after 45 years to 60′ checks (Fig. 3c). The C2 trough is more prominent in the pattern onset VEP waveform produced to smaller 15′ checks. C1 and C2 latencies are earlier to 15′ checks compared to 60′. The teenage pattern onset VEPs to 60′ were similar to those of the 20 year old shown in Fig. 3, a simple positive peak (Fig. 4).

**Fig. 5:**
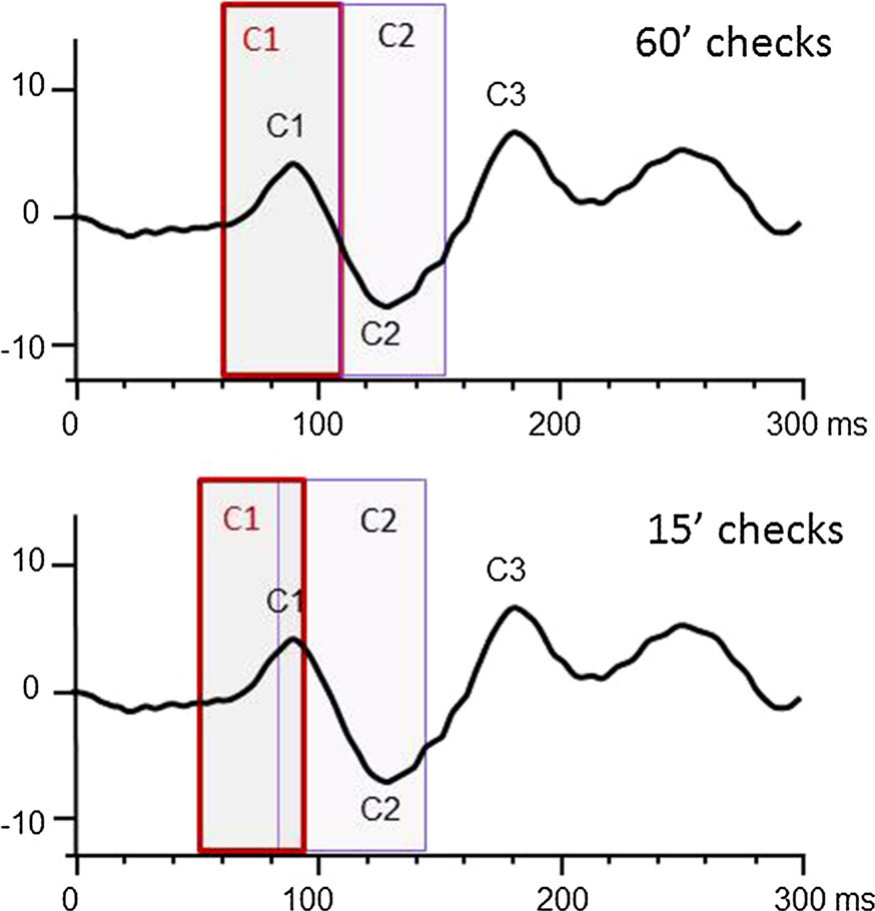
The latency range of the peaks C1 and C2 are displayed over the ISCEV waveform for 60′ and 15′ checks. The peaks to smaller checks are earlier than to larger checks. The ISCEV waveform peaks fall in the middle of the latency range of the adult C1 and C2 produced by 60′ check

The latency range of pattern onset VEP main peaks produced by 60′ and 15′ checks is detailed in Table 1. When the latency ranges are translated onto the standard ISCEV onset VEP waveform in Fig. 5, there is good agreement with the 60′ check values.

**Table 1:** The median and range of amplitude and latency of each peak of the pattern onset VEPs produced by 60′ and 15′ checks

	60′ median adult peaks (range)		15′ median adult peaks (range)	
C1	7uV@88 ms	(69–109 ms)	2uV@69 ms	(60–95 ms)
C2	11uV@109 ms	(109–150 ms)	14uV@90 ms	(79–143 ms)
C3	14uV@152 ms	(127–246 ms)	14uV@122 ms	(100–167 ms)

We also compared the onset waveforms from the same adults produced by the ISCEV standard field size 15° with those from checks presented with a slightly longer onset period of 230 ms in a larger 30° field. Though waveforms differed within the same individual to 60′ or 15′, we did not observe an intra-individual difference between the onset VEP waveforms produced when either of these checks was presented in 15° or 30° field, nor did we observe any differences in the onset VEP waveform elicited to 200 ms compared to 230 ms onset, nor when the inter-stimulus interval/offset interval was 330 ms cf 1000 ms.

## Discussion

Pattern onset stimulation is an essential VEP stimulus for paediatric clinics where defocus and nystagmus are often encountered. The ISCEV VEP standard recognises that inter-individual variability of VEP waveforms produced by onset and flash stimulation is high [1], but the within individual concordance means that an inter-ocular comparison of onset and flash VEP waveforms can provide valuable clinical information, as can an inter-ocular comparison of the trans-occipital distribution of monocular responses. Indeed onset stimulation is required for detection of chiasmal misrouting of albinism in older subjects [4–7].

Published illustrations most often show onset VEP waveforms with C1:C2:C3 ratios that resemble the ISCEV standard waveform example [1]. Our findings caution that the composition of the onset VEP waveform is dependent upon age and check size. Our data suggest the onset VEP waveform illustrated in the ISCEV VEP standard [1] most likely represents the response produced by 60′ pattern onset in a 45 year old subject. The onset VEP waveform produced by smaller 15′ checks has a more prominent C2, compared to 60′, and has earlier C1 and C2 latencies. The range of latencies for each component described in our study of ISCEV standard onset VEPs agrees well with other published adult work, e.g. C1 65–80 ms, C2 90–110 ms, C3 150–200 ms [8] and C1 80–110 ms [9].

Although the C1 component is better seen in older subjects, it may also be enhanced by rapid onset periods (e.g. 25 ms onset [10] or 40 ms onset [11] compared to ISCEV standard 200 ms), use of lateral electrodes and large checks [12]. Indeed the spatial tuning of adult onset VEP components is complex; for example, Kriss et al. [8] reported that C1 is largest to 72′, C2 largest to 9′ and C3 bimodally larger to 9′ and 110′.

The three peaks of the onset VEP, C1, C2 and C3, appear to represent an interaction and temporal summation of activity from different cortical sources. These multiple, simultaneously active areas are very close together. Our data suggest ageing differentially alters the relative contribution of one or each component to the summated onset VEP waveform, but it is challenge to attribute a specific peak to a cortical source. Researchers have sought innovative ways to solve this inverse problem [13]. Parametric manipulations that include contrast adaptation, localising stimulation to small quadratic fields, which show the dependence of the waveform on retinal location, and principal component analyses and coregistration of fMRI, EEG and MEG have been used to infer cortical dipoles [10, 14–17].

There is broad consensus that the onset VEP waveform has at least two overlapping time components: one from striate and the other from extra-striate areas [10, 14–17]. Classic studies from Jeffreys and Axford [10, 17] attributed C1 to striate cortex and C2 to extra-striate regions (for comparison C2 is P1 in di Russo et al. 2002 description [13]), whilst Spekreijse et al. [18] associated C1 with local luminance changes within the pattern arising from area 18 and C2, which is sensitive to contrast, defocus and pattern size, with striate areas [14]. This controversy is current some 50 years later [19, 20]. Mostly available data suggest C1 arises from multiple visual areas, but has a predominant contribution from V1 primary striate visual cortex in the early part of the waveform, whilst C2 reflects activity in dorsal and ventral extra-striate and C3 has also posterior parietal cortex contributions [21, 22].

During the early parametric studies two maturational phases for the onset VEP emerged: a rapid phase between birth and 8 months and followed by a slower phase ending at puberty with the C1–C2–C3 onset VEP morphology apparent at 16 years [11]. Published figures of paediatric pattern onset VEPs are very few. A population study of 214 children from 2 months to 12 years by De Vries-Khoe and Spekreijse [23] described how a negative peak (C2) became recognisable in the broad positive pattern onset VEP of children, with an incidence increasing continuously from 0% in the first five to ten months post-term to about 40% at 20 months of age and to approximately 100% around 8 years of age. Ossenblok et al. [24] confirmed this in a detailed study of equivalent dipole source localisation of 10 children aged 6–16 years and described the evolved response as being a positive peak at 130 ms preceded by a negativity at 100 ms. These changes in onset VEP waveform from childhood to adulthood were attributed to changes in the activity profile of the striate cortex, which dominates in younger children whilst extra-striate activity dominates in later life [16]. Apkarian and Tijsson [9] describe the maturation of the albino trans-occipital asymmetry with some waveform illustrations of paediatric onset VEPs. They argued that C2 is not developed because of the immaturity of the sensitivity to fine elements in the striate cortex, but show C2 is developed by 20 years (using 12′ checks and 40 ms onset). They highlighted that reliable contra-lateral asymmetry in the pattern onset VEP is most consistently seen in the C1 peak—as this is not apparent or well developed in children it helps explain why the flash VEP is a preferred stimulus for checking for albino misrouting in young children under 3 years. Lenassi et al. [25] plotted onset VEP data from 13 children, aged 1 year and less, and suggested mean C1 latency becomes ~120 ms at 6 months, with a range 75–155 ms at 6 years. This agrees with the wide range of peak onset VEP latency in our study of 16 children at 7 months of 76–134 ms. Although there is a preponderance of an early first peak, some waveforms are dominated by later positive peaks.

Surprisingly our study shows a third ‘maturational’ or differential phase in the onset VEP waveform, or rather continuous changes throughout adult life, with the emergence of a more prominent C1:C2 ratio with age. The underpinning physiological changes in the cortex between 40 and 60 years of age responsible for this are unclear and speculative. The occipital lobe is one of the brain areas most resilient to ageing. Myelination and synaptic pruning dominate the maturation changes in childhood whilst neuronal shrinkage and axonal fibre loss predominate ageing. Delineating a transition from maturation to degeneration associated with ageing is complicated to determine in vivo. Grey matter is fairly constant, but white matter volume increases until mid-40s, corresponding with a peak of myelination in some areas at 50 years, e.g. mesial temporal surface [26]. Brain weight is maximal around 20 years and does not reduce until after 50 years with a decline in brain volume starting around 45–50 years [26]. Age-related loss of grey matter is most prominent in the frontal and temporal lobes, with peak loss in dorsal brain areas around 50–70 years. The occipital lobes show least change, and although Good and colleagues [27] describe white matter loss in the occipital cortex, this occurs only towards the eighth and ninth decades. The network of higher-order regions that develop relatively late in adolescence shows accelerated degeneration in old age and heightened vulnerability to disorders that impact brain during adolescence and ageing [28, 29]. According to this ‘last in first out’ theory, we may speculate that that the onset components associated with extra-striate areas will be affected first by ageing and may underpin the waveform changes we have described.

In terms of VEP generation, these gradual anatomical and physiological changes in cortical tissue could alter extra-cellular or intra-cellular resistance, which in turn may change the relative amplitude and/or timing of one of the components contributing to the summated pattern onset VEP signal. A small latency difference in one component can have a substantial impact on the summated waveform shape, as seen when a ‘negative’ prolonged on flash ERG is modelled by delaying by 5 ms the depolarising bipolar contribution to the photopic prolonged on off macaque ERG a-wave [30, 31].

In summary, our data illustrate the changes in the ISCEV standard pattern onset VEP waveform throughout life and provide a template for clinical comparison. Further studies are needed to understand whether these changes may be exploited to explore ageing mechanisms and vulnerability to degenerative disease.
